# Drought-Stress-Induced Changes in Chloroplast Gene Expression in Two Contrasting Strawberry Tree (*Arbutus unedo* L.) Genotypes

**DOI:** 10.3390/plants12244133

**Published:** 2023-12-11

**Authors:** João Martins, Mariana Neves, Jorge Canhoto

**Affiliations:** Centre for Functional Ecology, Associate Laboratory TERRA, Department of Life Sciences, University of Coimbra, Calçada Martim de Freitas, 3000-456 Coimbra, Portugal; mariananevespt@gmail.com (M.N.); jorgecan@uc.pt (J.C.)

**Keywords:** chlorophyll, net CO_2_ assimilation, photosynthetic activity, PSI/PSII, strawberry tree

## Abstract

This study investigated the effect of drought stress on the expression of chloroplast genes in two different genotypes (A1 and A4) of strawberry tree plants with contrasting performances. Two-year-old plants were subjected to drought (20 days at 18% field capacity), and the photosynthetic activity, chlorophyll content, and expression levels of 16 chloroplast genes involved in photosynthesis and metabolism-related enzymes were analyzed. Genotype-specific responses were prominent, with A1 displaying wilting and leaf curling, contrasting with the mild symptoms observed in A4. Quantification of damage using the net CO_2_ assimilation rates and chlorophyll content unveiled a significant reduction in A1, while A4 maintained stability. Gene expression analysis revealed substantial downregulation of A1 (15 out of 16 genes) and upregulation of A4 (14 out of 16 genes). Notably, *psbC* was downregulated in A1, while it was prominently upregulated in A4. Principal Component Analysis (PCA) highlighted genotype-specific clusters, emphasizing distinct responses under stress, whereas a correlation analysis elucidated intricate relationships between gene expression, net CO_2_ assimilation, and chlorophyll content. Particularly, a positive correlation with *psaB*, whereas a negative correlation with *psbC* was found in genotype A1. Regression analysis identified potential predictors for net CO_2_ assimilation, in particular *psaB*. These findings contribute valuable insights for future strategies targeting crop enhancement and stress resilience, highlighting the central role of chloroplasts in orchestrating plant responses to environmental stressors, and may contribute to the development of drought-tolerant plant varieties, which are essential for sustaining agriculture in regions affected by water scarcity.

## 1. Introduction

Drought stress is one of the most significant environmental factors affecting plant growth and productivity worldwide, causing major reductions in crop yield and affecting the sustainability of agriculture [1]. In recent years, increasing climate variability and global warming have led to more frequent and severe drought events globally [2], making it crucial to understand the mechanisms that plants use to cope with water deficit conditions. Plant resistance to drought stress is complex and involves various mechanisms, including changes in gene expression [3]. Plant genes that are differentially expressed under drought stress are involved in several biological processes, such as photosynthesis, water transport, osmotic regulation, and stress response [3]. Therefore, the study of gene expression in plants under drought stress can provide valuable insights into the molecular mechanisms involved in plant adaptation to drought.

One approach to understanding plant molecular mechanisms in response to drought stress is to investigate the expression patterns of genes involved in specific metabolic pathways. Although several studies have been conducted to evaluate the expression patterns of nuclear genes [4,5], very few have been focused on the expression of chloroplast genes. As chloroplasts are dynamic organelles capable of fine-tuning their gene expression in response to changing environmental conditions [6,7], drought stress triggers a cascade of events within the chloroplasts, leading to changes in transcript abundance, protein expression, and ultimately, cellular changes [8]. For instance, a downregulation of transcripts related to photosynthesis was found in a maize (*Zea mays* L.) inbred line under moderate water deficit [9]. The chloroplast proteome of both tomato (*Solanum lycopersicum* L.) and common bean (*Phaseolus vulgaris* L.) revealed that the proteins involved in photosynthesis were largely affected under water stress [10,11]. Thus, understanding the specific responses of chloroplast-encoded genes and the regulatory networks regulating their expression in strawberry tree (*Arbutus unedo* L.) will provide insights into the molecular basis of its drought stress adaptation.

Strawberry tree, a member of the Ericaceae family, is a Mediterranean evergreen tree [12] known for its ability to colonize marginal lands and regenerate after fire, making it a perfect plant for reforestation programs, besides numerous economical applications [13]. Its adaptation to diverse habitats and climatic conditions also makes it an excellent model system and has garnered interest in understanding its stress response mechanisms. Strawberry tree holds considerable ecological and economic importance in Portugal [13]. Thus, understanding the impact of drought stress on this species is crucial, as it aligns with the country’s agricultural sector, including forestry, fruit production, and biodiversity conservation. Furthermore, studying the impact of drought stress on local flora like strawberry tree contributes to our understanding of plant resilience in the challenging environmental conditions the Mediterranean region has experienced, with pronounced shifts in climate patterns, marked by recurring severe drought events over recent decades and an expected future decline in precipitation [14,15]. Previous studies focused on physiological responses to drought stress underline the water use conservation strategy of strawberry tree via stomatal closure, typical of isohydric species [16,17]. More recent studies have shown the relevance of the plant genotype under water deficit conditions and pinpointed metabolomic shifts in response to stress [18,19,20]. However, the intricate molecular mechanisms controlling plant response to water deficit remain largely unexplored. Understanding the response of chloroplast genes to drought stress in strawberry tree plants can provide valuable information on the molecular mechanisms involved in plant adaptation to changing environmental conditions.

Thus, in this study, to gain insights into the molecular mechanisms involved in plant adaptation to water deficit conditions, the effect of drought stress on chloroplast gene expression in two different genotypes of strawberry tree plants with contrasting performances was investigated. For this purpose, two genotypes (A1 and A4) were selected due to contrasting tolerance to drought, based on physiological and metabolomic data [20]. By analyzing the expression levels of 16 chloroplast genes involved in photosynthesis and energy-metabolism-related enzymes, and correlating them with photosynthetic parameters, we aimed to determine how strawberry tree genotypes with a contrasting performance under drought respond at the molecular level. The results of this study provide valuable information on the molecular mechanisms involved in plant adaptation to drought stress and may contribute to the development of drought-tolerant plant varieties, which are essential for sustaining agriculture in regions affected by water scarcity.

## 2. Results

To investigate the molecular response of strawberry tree to drought stress, two-year-old plants were subjected to drought (20 days at 18% field capacity), and the photosynthetic activity, chlorophyll content, and expression of several chloroplast genes were analyzed. 

### 2.1. Photosynthetic Activity and Chlorophyll Content

**Figure 1:**
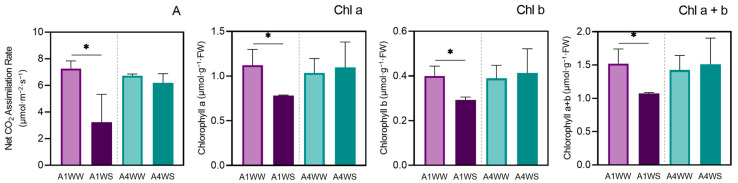
Drought stress effects on net CO_2_ assimilation (A) and chlorophyll content (Chl) in genotypes A1 and A4. In genotype A1, drought stress significantly reduced net CO_2_ assimilation (more than 50%) and chlorophyll content (Chl a, Chl b, and Chl a + b), while genotype A4 remained largely unaffected. Means ± SDs, *n* = 3, * indicate significant differences between treatments for each genotype at *p* ≤ 0.05 according to a Student’s *t*-test. WW—well watered (control), WS—water stress.

After 20 days under drought, the plants from genotype A1 showed clearly visible wilting phenotypes as well as curling leaves. On the other hand, the plants from genotype A4 showed only mild symptoms caused by drought stress. The degree of damage to the plants was quantified by measuring the net CO_2_ assimilation rate (A) and chlorophyll content (Chl a, Chl b, and Chl a + b). The results showed that the net CO_2_ assimilation rate greatly decreased (more than 50%) after the stress imposed in genotype A1, whereas no variation was observed in genotype A4 (Figure 1). Like net CO_2_ assimilation, the chlorophyll content (Chl a, Chl b, and total Chl) in the leaves of the strawberry tree plants decreased in genotype A1 when the control and stressed plants were compared, and no difference was found in genotype A4 (Figure 1). 

### 2.2. Chloroplast Gene Expression

**Figure 2:**
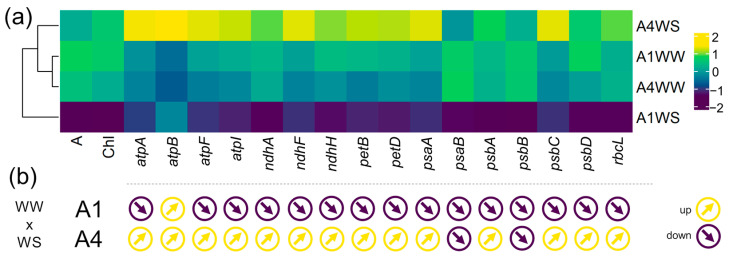
Drought stress effects on chloroplast gene expression in genotypes A1 and A4: (**a**) heatmap with relative gene expression, net CO_2_ assimilation, and total chlorophyll, with built-in dendrogram, highlighting the clustering of gene expression patterns under different conditions; (**b**) arrows pointing up or down indicate whether a gene is upregulated or downregulated. WW—well watered (control), WS—water stress.

To further determine the effects of drought stress on the expression of strawberry tree chloroplast genes, 16 chloroplast genes that are involved in photosynthesis (Table S1) were selected, and the transcript levels of these chloroplast genes were analyzed using qPCR. The results showed that most of the genes analyzed were downregulated by drought in genotype A1 (15 out of 16) and upregulated in genotype A4 (14 out of 16) (Figure 2a,b, Table S2). This pattern can clearly be observed in the heatmap (Figure 2a), and a clustering analysis grouped the control groups (A1WW and A4WW), whereas the stress treatments (A1WS and A4WS) are clustered separately. The gene *psbC* was markedly downregulated in genotype A1 upon drought treatment with a fold change (FC) of 0.029, followed by *psbD* (FC = 0.052) and *atpA* (FC = 0.061) (Table S2). The expression of *psbB* and *atpF* also decreased considerably in this genotype (FC = 0.222 and 0.246, respectively). In the opposite direction, the expression of *atpA*, *atpB*, *atpF*, and *psbC* greatly increased under drought in genotype A4 (FC = 3.305, 2.479, 2.596, and 2.985, respectively) (Table S2).

**Figure 3:**
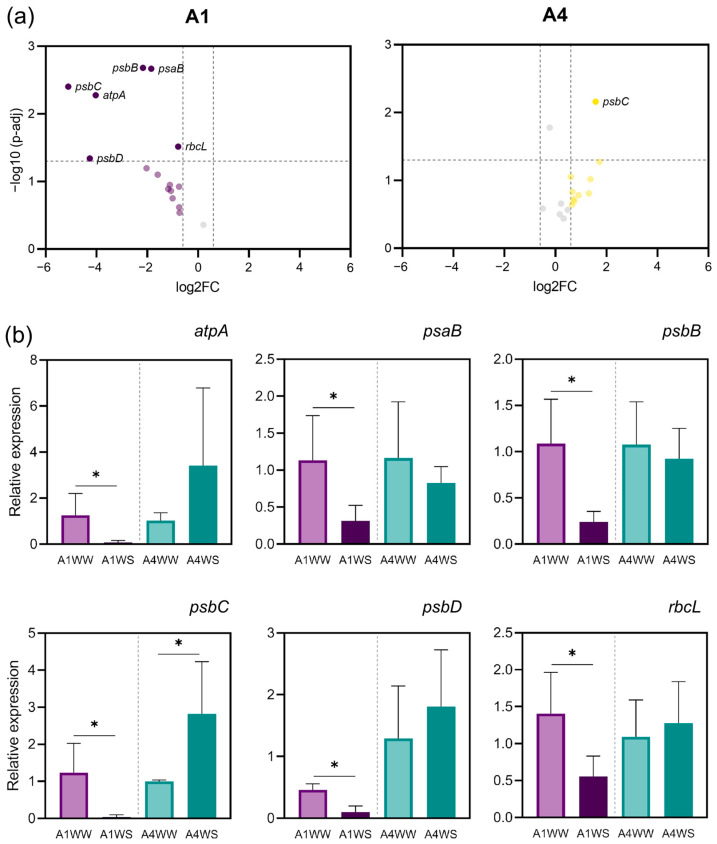
Differentially expressed genes in genotype A1 and A4 under drought stress: (**a**) volcano plot depicting the log2 fold change in gene expression (*x*-axis) and the negative logarithm (base 10) of the *p*-values (*y*-axis). Genes with significant downregulation (*p* < 0.05) are highlighted with purple data points, including *atpA*, *psaB*, *psbB*, *psbC*, *psbD*, and *rbcL*, whereas genes highlighted with yellow data points (*psbC*) are upregulated (*p* > 0.05); (**b**) bar plots illustrating the relative expression levels of the differentially regulated genes in genotype A1 and A4. Genes *atpA*, *psaB*, *psbB*, *psbD*, and *rbcL* exhibit significantly reduced expression compared to control conditions, whereas *psbC* shows a reduced expression in genotype A1 and increased in genotype A4. Means ± SDs, *n* = 3, * indicate significant differences between treatments for each genotype at *p* ≤ 0.05 according to Student’s *t*-test. WW—well watered (control), WS—water stress. Genes in grey in the volcano plot have −0.6 ≥ log2FC ≤ 0.6.

Volcano plots were constructed to visualize the differential expression of genes. In genotype A1, several genes displayed notable downregulation under drought stress conditions. Specifically, genes *atpA*, *psaB*, *psbB*, *psbC*, *psbD*, and *rbcL* exhibited statistically significant decreases in expression levels (*p* < 0.05) (Figure 3a). The most prominent among these was *psbC*, which showed a marked reduction in expression levels (log2 fold change < −5.0). Conversely, within genotype A4, the panorama was clearly distinct. From all the genes, *psbC* emerged as exhibiting a statistically meaningful upregulation in response to drought stress.

Bar plots were generated to provide a detailed view of the relative expression levels of the differentially regulated genes in both genotypes. As mentioned before, in genotype A1, the downregulated genes, *atpA*, *psaB*, *psbB*, *psbC*, *psbD*, and *rbcL*, exhibited significantly reduced expression levels compared to in the control conditions (Figure 3b). Notably, *psbC* displayed the most substantial decrease in expression in genotype A1. Conversely, in genotype A4, only *psbC* was observed to be upregulated. These results underscore the differential response of the two genotypes to drought stress, with genotype A1 exhibiting a more pronounced downregulation of multiple genes, including *psbC*, compared to A4. The differences in gene expression patterns between the two genotypes are evident, with *psbC* being the only gene that responds positively to drought stress in genotype A4.

**Figure 4:**
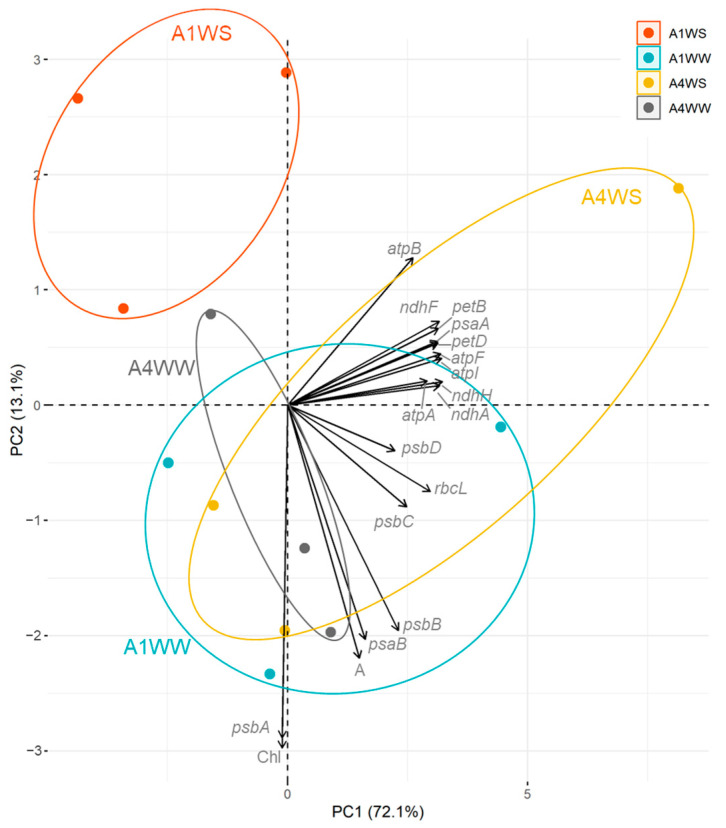
Principal Component Analysis (PCA) of gene expression, net CO_2_ assimilation rate, and chlorophyll content. PC1 and PC2 capture 83.3% of the dataset’s variability, with genotypes A1 and A4 under well-watered conditions clustering closely, while A1 under water stress is distinctly separated, primarily along PC1. WW—well watered (control), WS—water stress.

A Principal Component Analysis (PCA) was conducted on the gene expression dataset to unveil the inherent patterns and relationships among different genotypes and their respective watering conditions. The first two principal components, PC1 and PC2, accounted for a substantial portion of the overall variance in the dataset (Figure 4). PC1 explained 65.1% of the total variance, while PC2 captured an additional 18.2%. Together, these two components elucidated 83.3% of the dataset’s variability, emphasizing their significance in summarizing the gene expression profiles. Distinct clusters emerged on the PCA plot, reflecting the shared characteristics among data points: notably, cluster 1 (A1WW, A4WW, and A4WS) with genotypes A1 and A4 under well-watered (WW) conditions and A4 under water stress (WS) exhibited a close grouping. This clustering suggests that these three groups share similar gene expression patterns that manifest consistently across their respective conditions. On the other hand, cluster 2 (A1WS) data points, representing genotype A1 under water stress conditions, were distinctly separated from the other cluster. This separation highlights that the gene expression patterns of A1 under water stress are notably distinct from the other conditions. PC1, capturing the most significant source of variability, was pivotal in differentiating A1 under water stress from other conditions. The separation along PC1 emphasizes that this component captures the key variations contributing to the differences between A1 under water stress and the other genotypes.

### 2.3. Correlation between Net CO_2_ Assimilation, Chlorophyll Content, and Gene Expression

**Figure 5:**
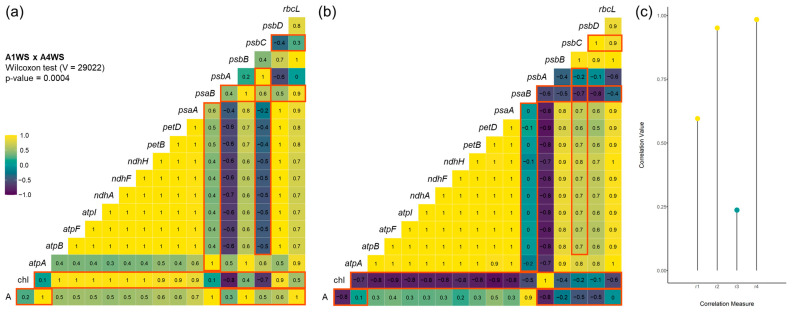
Correlation analysis between gene expression, net CO_2_ assimilation, and chlorophyll, in genotypes A1 and A4 under drought: (**a**) correlation analysis for genotype A1 under drought; (**b**) correlation analysis for genotype A4 under drought; (**c**) similarity measure between correlation matrices. WS—water stress. Most significant differences between the correlograms are highlighted in red. In (**c**) yellow dots represent correlation values ≥ 0.5 and green dots < 0.5.

Correlation analysis was conducted to elucidate the intricate relationships between gene expression patterns, net CO_2_ assimilation, and chlorophyll content. A cluster of genes, namely, *atpA*, *atpB*, *atpF*, *atpI*, *ndhA*, *ndhF*, *ndhH*, *petB*, *petD*, *psaA*, *psbB*, *psbD*, and *rbcL*, exhibited remarkably strong positive correlations in both genotypes under stress, with correlation coefficients of 1 in most cases (Figure 5a,b). Among the genes displaying robust correlations with one another, a positive correlation emerged with *psaB*, whereas a negative correlation was found with *psbC* in genotype A1. In contrast, the correlation between the atp, ndh, and pet clusters with *psaB* and *psbC* is 0 and positive, respectively, in genotype A4. A similar observation was made for the correlation between chlorophyll content (Chl) and most of the genes, with a positive correlation in most cases for genotype A1, whereas a negative correlation was obtained in genotype A4. Similarly, a positive correlation between the net CO_2_ assimilation rate and most genes was found in genotype A1, while, in genotype A4, the correlation was only slightly positive or negative for some genes. This difference is more evident for the genes *psbA* (A1: 0.3, A4: −0.8), *psbB* (1, −0.2), *psbC* (0.5, −0.5), *psbD* (0.6, −0.5), and *rbcL* (1, 0). The similarity measures used to compare the correlation matrices suggest a substantial linear relationship between the matrices (r1 = 0.596) (Figure 5c). Furthermore, the r2 (0.951) and r4 (0.984) values suggest that the matrices have very similar structural and dominant patterns. On the other hand, the very low r3 value (0.237) indicates that the orientation of the principal component directions is not aligned. Overall, the combination of these results indicates the structural patterns in the matrices are very similar, but their dominant directions (as assessed via r3) are not closely aligned, suggesting that while the relationships between the variables are similar, the dominant patterns themselves differ in their orientations. Although strong similarities emerged when the two correlations were compared, a statistically significant difference was found between the correlations of genotype A1 and A4 under stress (*p*-value = 0.0004).

### 2.4. Predictive Models of Drought Tolerance

**Figure 6:**
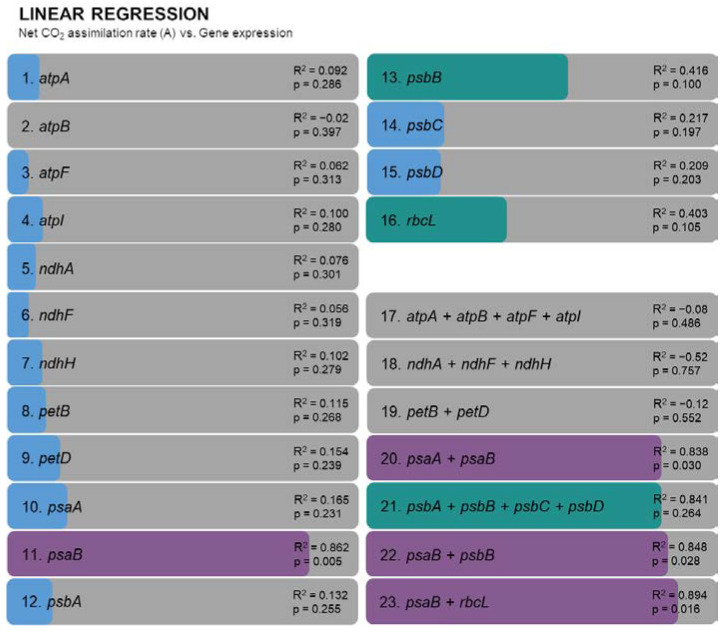
Regression models based on relative gene expression to predict net CO_2_ assimilation rates: R^2^ of linear regression analysis with *p*-values. Different colors indicate the confidence of the model: purple—models with R^2^ > 0.8 and *p*-value < 0.05, green—R^2^ > 0.4 and *p*-value > 0.05, blue—R^2^ < 0.4 and *p*-value > 0.05.

Regression analysis was employed to uncover the underlying relationships among the gene expression levels and explore potential predictive models based on the obtained data. The adjusted R-squared (R^2^) values for individual genes elucidate their respective contributions to predicting net CO_2_ assimilation rates. Among these, the gene *psaB* stands out with a relatively high R^2^ value of 0.862 (*p* = 0.005) (Figure 6, Table S3). Multiple linear regression models were constructed to investigate the combined effects of gene clusters. Notably, the model encompassing *psaA* and *psaB* (psa cluster) showcased a high R^2^ value of 0.838, albeit with a *p*-value of 0.030. Similarly, the combination of *psaB* and *psbB*, as well as *psaB* and *rbcL*, provides models with a high R^2^ of 0.848 (*p* = 0.028) and 0.894 (*p* = 0.016), respectively, hinting at potentially promising predictive models. On the other hand, the atp (*atpA*, *atpB*, *atpF*, and *atpI*), ndh (*ndhA*, *ndhF*, and *ndhH*), and pet clusters (*petB* and *petD*) exhibited very low R^2^ values, suggesting a low predictive capacity. Finally, and despite a higher R^2^ value being obtained with the psb cluster (*psbA*, *psbB*, *psbC*, and *psbD*), the predictors in this model are not statistically significant (*p* = 0.264).

## 3. Discussion

Drought stress represents a significant and pervasive challenge for agriculture, diminishing crop productivity and quality. *A. unedo* assumes great ecological and economic relevance as a valuable fruit-bearing species, especially in regions prone to water scarcity. To address the pressing need for drought-resilient genotypes and to gain a comprehensive understanding of their responses to drought stress at various levels, this study investigates two contrasting genotypes.

### 3.1. Photosynthetic Activity and Chlorophyll Content

The convergence of net CO_2_ assimilation, chlorophyll content, and gene expression analyses provides a profound understanding of genotype-specific responses to water stress. Using a multidimensional analysis, a comprehensive narrative emerges that sheds light on the intricate biochemical and physiological mechanisms underlying stress adaptation. The observed reduction in net CO_2_ assimilation and chlorophyll content, specifically within genotype A1 under stress conditions, is in accordance with previous results [20]. In contrast, genotype A4 was able to maintain CO_2_ assimilation unchanged upon drought. These results correlate with chloroplast gene expression patterns, as a general downregulation was observed in genotype A1, whereas an upregulated pattern was observed in genotype A4. The alignment of these results with data from a previous metabolomic study [20], i.e., a specific metabolomic profile associated with genotype A4, underscores the robustness of the observed phenomenon and suggests an intricate interplay between gene expression, metabolite profiles, and physiological responses that fine-tunes the plant’s response to water scarcity. As has been shown before, the reduction in photosynthetic yield is not due to limitation in stomatal function but rather to the reduced carboxylation efficiency, as the intercellular CO_2_ concentration only slightly decreases upon drought [20]. Thus, the unchanged net CO_2_ assimilation and chlorophyll content in genotype A4 under water stress could be associated with the metabolite shifts detected earlier, and an integrated view suggests that metabolic reconfiguration and gene expression modulation act in concert to optimize resource allocation and energy use under stress condition.

### 3.2. Chloroplast Gene Expression

Gene expression analysis of the key components of the photosynthetic apparatus, such as PSI, PSII, cytochrome, NADH dehydrogenase, ATP synthase, and RuBisCO, provides an understanding of the molecular mechanisms of how plants cope with drought stress. The use of different genotypes with contrasting drought tolerance offers great insights into drought effects and may contribute to identifying putative candidate genes involved in the plant response to water stress. The gene downregulation found in genotype A1 under water stress agrees with previous results in *Arabidopsis thaliana*, where prolonged drought stress reduced the chloroplast-encoded gene transcript levels [21]. Downregulation of the *psaB*, *psbB*, and *psbC* genes was also found in later periods of water deficit in maize [9]. Similarly, in *Vitis vinifera*, the CO_2_ assimilation rate decreased after 20 days of drought treatment together, with a downregulation of genes related to PSI, PSII, and cytochrome b6-f complex, including *psaB*, *psbB*, and *psbC* [22]. Drought stress also downregulates *rbcL* gene expression and other photosynthesis-related genes in *Paeonia lactiflora* [23]. A decrease in the *rbcL* gene expression was also observed in *Pinus halepensis* after drought [24]. The downregulation of the *atpA* and *rbcL* genes found in genotype A1 suggests a decrease in both protein expression and the activity of ATP synthase and RuBisCO. A downregulation of these protein expressions has already been described in a drought-susceptible rice cultivar [25]. A decrease in the abundance of ATP synthase alpha subunits was also found in *Phaseolus vulgaris* under drought stress [11,26]. The altered gene expression profile observed in our study, together with the reduced net CO_2_ assimilation and chlorophyll content, suggests a reduced photosynthetic capacity in genotype A1 under water stress, explaining its greater susceptibility. The upregulation or stability of gene expression in genotype A4 suggests a compensatory mechanism related to energy production under drought stress, resulting in greater plant tolerance.

The decrease observed in the relative expression of PSII genes (3 out of 4: *psbB*, *psbC*, and *psbD*) in genotype A1, seems to indicate that under drought, PSII is more affected than PSI (1 out of 2 decreases: *psaB*). In *A. thaliana*, although drought affects both photosystems, PSII is more affected at an early stage, whereas damage in PSI can be observed at a later stage [27], suggesting more prolonged stress might cause a similar effect in *A. unedo*. Although most psa and psb genes were upregulated in genotype A4, *psbC* showed the highest increase. Its upregulation might be particularly important for maintaining the stability and function of PSII under drought stress because *psbC* encodes CP43, a core subunit of the PSII reaction center, which is essential for stabilizing the oxygen-evolving complex and intervenes in the PSII assembly [28]. Interestingly, *psbB* encodes CP47, a closely related chlorophyll-binding protein [29], but its expression is downregulated in both genotypes, suggesting CP43 (encoded by *psbC*) might play a particular role in drought tolerance mechanisms. In fact, it has been suggested that CP43 could provide a distinct role from the D1 polypeptide and could serve as the sole amino acid ligand for the oxygen-evolving Mn_4_Ca complex [28].

### 3.3. Correlation between Net CO_2_ Assimilation, Chlorophyll Content, and Gene Expression

The observed high correlations within specific gene clusters, coupled with their interactions with photosystem-related genes, photosynthetic parameters, and chlorophyll content, underscore a comprehensive network of synchronized responses to water stress. This intricate network potentially facilitates precise adjustments within the photosynthetic machinery, optimizing energy usage, water efficiency, and CO_2_ assimilation. The strong positive correlations found between atp, ndh, pet, *rbcL*, and some photosystem genes (*psaA*, *psbB*, and *psbD*) in both genotypes suggests a tightly orchestrated regulatory framework governing these genes that may play a pivotal role in responses to water deficit. In contrast, the correlation between the atp, ndh, and pet clusters with *psbC* is negative in genotype A1 and positive in A4. In a sensitive genotype like A1, this negative correlation may indicate that the plant is struggling to maintain energy production and photosynthetic activity, and the downregulation of these clusters might reflect a response to conserve energy and resources to cope with the adverse conditions. On the other hand, the response of A4 under stress suggests that this genotype is more effective at maintaining energy production and photosynthesis even in challenging conditions and continues to support the plant’s survival and growth. Notably, the negative correlation of most genes with *psbA* in both genotypes suggests a potential regulatory mechanism that modulates *psbA* expression in response to water stress. This modulation could possibly fine-tune the balance between photosynthetic light capture and energy utilization, as *psbA* encodes the D1 reaction center protein of photosystem II and is continuously subjected to photodamage [7]. Thus, a continuous drought stress may activate a compensatory mechanism leading to de novo synthesis of this protein, which would be essential for repairing PSII photodamage.

The correlations between gene expression, net CO_2_ assimilation, and chlorophyll content further enrich the narrative. The positive correlations between these factors and key photosystem genes (*psaB*, *psbB*) seems to accentuate their central roles in photosynthetic efficiency. Although the *rbcL* gene encodes a key enzyme to the carbon fixation process during photosynthesis (RuBisCO), the absence of a correlation (r = 0) between the net CO_2_ assimilation and *rbcL* gene expression in genotype A4 can be attributed to the complex interplay of various factors influencing carbon assimilation in the plant. Firstly, in some cases, impairment of RuBisCO does not influence photosynthesis until a very severe level of drought [30]. Furthermore, regulatory mechanisms, such as post-translational modifications, protein–protein interactions, and the active/inactive state of the enzyme, can also significantly impact the actual activity of RuBisCO [31]. Consequently, the gene expression level alone may not fully reflect the enzyme’s functionality or the net CO_2_ assimilation rate. A negative correlation was also found between *rbcL* and chlorophyll content in genotype A4, caused by a great increase in *rbcL* expression levels and steady-state chlorophyll. Although this scenario may appear counterintuitive, a potential explanation for this negative correlation could be linked to the high nitrogen requirements for the synthesis of the RuBisCO protein (approximately 20% of leaf nitrogen) when the expression of *rbcL* increases, leading to a decrease in the availability of nitrogen for chlorophyll production, which is a nitrogen-containing pigment [32]. This could also explain the negative correlation between chlorophyll content and the expression of most genes observed in genotype A4. Interestingly, the chlorophyll content exhibited a strong positive correlation (r = 1) with *psbA* in genotype A4, and a negative correlation (r = −0.8) in genotype A1. This could imply that *psbA* plays a significant role in governing chlorophyll content under the studied conditions, as the levels of chlorophyll are reduced in genotype A1. In fact, in *Nicotiana tabacum*, the overexpression of *psbA* gene from maize resulted in plants with a higher drought tolerance and also higher levels of chlorophyll [33]. Furthermore, *psbA* deletion mutants showed a disrupted redox state, with a decrease in antioxidant enzymes [34], which might accelerate chlorophyll decomposition [23].

### 3.4. Predictive Models of Drought Tolerance

The regression analysis provides an understanding of how gene expression levels may contribute to the observed outcomes, specifically carbon assimilation. As expected, satisfactory linear models emerge as potentially predictors from some of the genes found to be downregulated only for the sensitive genotype (A1), particularly genes like *psaB*, *psbB*, and *rbcL*. A previous work on *A. unedo* successfully deployed machine learning approaches to predict complex phenotype traits [20], as the concentration of certain metabolites were found to be good predictors of the net CO_2_ assimilation rates in plants under drought. While this analysis has provided significant insights, the *p*-values associated with some models suggest a degree of uncertainty. Thus, further validation is warranted to solidify the predictive capacity of these models, by expanding the dataset and considering other variables that could yield a more comprehensive understanding of the gene–environment interactions.

Collectively, the findings presented in this study paint a comprehensive picture of how chloroplast gene expression plays a pivotal role in plant responses to water stress. Genotype-specific responses are underlined, emphasizing how chloroplast gene expression patterns, metabolite changes, and physiological responses collectively mold the distinctive adaptive strategies of each genotype. Correlation analysis and the gene network highlight the significant impact of gene expression patterns on genotype responses and the strong correlations with key photosystem genes underscore their central roles in maintaining photosynthetic efficiency. Moreover, the results from regression analysis emphasize the importance of gene clusters and combinations, guiding the construction of predictive models that bridge the molecular mechanisms and observed outcomes.

## 4. Conclusions

In conclusion, the integrated analyses presented in this study offer comprehension of the genetic, physiological, and predictive dimensions of plant responses to water stress. This approach advances our understanding of the intricate interactions between chloroplast gene expression, photosynthesis, and predictive modeling, ultimately contributing to the development of strategies for enhancing stress tolerance and crop performance within agricultural contexts.

However, to develop a full picture of the resistance mechanism to drought at the chloroplast level, additional studies will be needed that consider the involvement of extrinsic proteins, such as PsbO, PsbP, PsbQ, and PsbR, as their involvement in PSII efficiency and the repair system has been reported [35,36]. This study underscores the significant role of chloroplasts in responding to environmental stress and stimuli, which is evident in the differential expression of genes: 15 out of 16 were downregulated by drought in genotype A1, whereas 14 out of 16 were upregulated in genotype A4.

Future research might explore the interconnections between splicing events, transcript stability, the involvement of extrinsic proteins, and the links between chloroplasts and nuclear genes, in order to uncover novel genetic targets and mechanisms essential for enhancing plant resilience in the face of changing environmental conditions.

## 5. Materials and Methods

### 5.1. Plant Material and Drought Stress Assay

Two distinct genotypes, originating from different regions with varying drought tolerance profiles, were chosen for investigation: genotype A1 hails from the central area of Portugal, characterized by an average annual rainfall exceeding 1000 mm, but displaying limited drought tolerance. On the other hand, genotype A4 originates from the southern region, with an average yearly rainfall below 500 mm, showcasing significant drought tolerance. The evaluation of genotype responses to drought stress was conducted based on the prior physiological and metabolomics data previously reported [20]. Genotype A1 was established in vitro from a mature tree, while genotype A4 was initiated from a seedling, following the methodology previously described [18]. To initiate axillary shoot proliferation, the shoots were cultured in Anderson Rhododendron medium [37] supplemented with 6-benzylaminopurine (BAP; 8 µM; Sigma-Aldrich, St. Louis, MO, USA), sucrose (3% *w*/*v*, Duchefa, Haarlem, The Netherlands), and agar (0.6% *w*/*v*, Duchefa). The medium’s pH was balanced to 5.7 followed by autoclaving at 121 °C for 20 min (resulting in a gel strength of 800–1100 g·cm^−2^ post-autoclaving). The cultivation was carried out in Microbox plastic containers (O118/80 + OD118 with white filter, SacO_2_, Deinze, Belgium) containing 100 mL of medium. The growth chamber conditions included a 16 h photoperiod, light irradiance of 15–20 µmol·m^−2^·s^−1^ (LED lamps), and a temperature of 25 °C, with cultivation cycles spanning 8 weeks.

For the rooting stage, 3 cm long shoots were immersed in a solution of indole-3-butyric acid (IBA; 10 µM; Sigma-Aldrich) for 30 s and positioned in covered containers with perlite (Siro, Mira, Portugal). These containers were placed within a walk-in growth chamber (FitoClima 10000 HP, Aralab, Rio de Mouro, Portugal) set to a 16 h photoperiod at 25 °C, with 70% humidity and 250 µmol·m^−2^·s^−1^ irradiance. The cover was gradually lifted, and after a month, the plants were transplanted into individual containers (1.700 cm^3^) filled with a substrate composed of peat (30-0; Siro) and perlite (3:1; *v*/*v*). These plants were maintained in these conditions for a period of two years, with regular watering to maintain 70–80% of field capacity. Subsequent to this period, the plants were subjected to two distinct watering regimes employing the gravimetric approach: WW—representing well-watered conditions (with watering adjusted to reach 70% of field capacity), and WS—representing water stress conditions (with watering adjusted to achieve 18% of field capacity) [20]. After 20 days under these conditions, the apical leaves from three plants per genotype per treatment were collected, flash-frozen in liquid nitrogen, ground with mortar and pestle, and stored at −80 °C until further processing.

### 5.2. Photosynthetic Activity and Chlorophyll Content

#### 5.2.1. Photosynthetic Activity

In situ measurement of the net CO_2_ assimilation rate was performed using a portable infrared gas analyzer (LCpro+, ADC, Hoddesdon, UK), operating in open mode and under the following conditions: photosynthetic photon flux density: 350 µmol·m^−2^·s^−1^; air flux: 200 mol·s^−1^; block temperature: 25 °C; and atmospheric CO_2_ and H_2_O concentration. Data were recorded when the measured parameters were stable (2–6 min).

#### 5.2.2. Chlorophyll Content

The total chlorophyll in the extracts was estimated according to Sims and Gamon [38]. Briefly, 50 mg of frozen plant material was ground in 2 mL of acetone:Tris buffer, 50 mM, pH 7.8 (80:20), and centrifuged for 5 min (10,000× *g*, 4 °C). The supernatant was collected and extraction was repeated with 3 mL acetone:Tris. Finally, acetone:Tris was added to the supernatants to obtain a final volume of 6 mL. Samples were kept on ice and protected from light during the entire process. The absorbance of the supernatants was read at 470 nm, 537 nm, 647 nm, and 663 nm on a UV-Vis spectrophotometer. Acetone:Tris buffer, 50 mM, pH 7.8 (80:20), was used as blank. The chlorophyll a (Chl a) and chlorophyll b (Chl b) contents were calculated according to the following equations: Chla = 0.01373 × A663 − 0.000897 × A537 − 0.003046 × A647; Chlb = 0.02405 × A647 − 0.004305 × A537 − 0.005507 × A663.

### 5.3. Chloroplast Gene Expression

#### 5.3.1. RNA Extraction and cDNA Synthesis

Before RNA extraction, 1 mL of sorbitol wash buffer (100 mM Tris-HCl pH 8.0 (Sigma-Aldrich), 0.35 M sorbitol (Sigma-Aldrich), 5 mM EDTA pH 8.0 (Sigma-Aldrich), 1% (*w*/*v*) polyvinylpyrrolidone (M_W_: 40,000, Sigma-Aldrich), and 1% (*v*/*v*) 2-mercaptoethanol (Sigma-Aldrich) added just before extraction) was added to 50 mg of macerated plant material, mixed using a vortex, and centrifuged at 5000× *g* for five minutes at room temperature. The supernatants were discarded and the total RNA was extracted using a Quick-RNA Miniprep Kit (Zymo Research, Irvine, CA, USA) following the supplier’s instructions. The final concentration of RNA of each sample was measured using a spectrophotometer (NanoDrop One, Thermo Scientific, Waltham, MA, USA) and its purity was confirmed using the A260/A280 and A260/A230 ratios. The RNA integrity was further validated using the Qubit™ RNA IQ Assay Kit (Invitrogen™, Thermo Fisher Scientific, Waltham, MA, USA). First-strand cDNA was generated using the NZY First-Strand cDNA Synthesis Flexible Pack (NZYTech, Lisbon, Portugal) according to the manufacturer’s instructions, from 1 μg of total RNA from 3 biological replicates for each treatment and genotype.

#### 5.3.2. qPCR

Quantitative PCR gene expression analysis of 16 genes coding for ATP synthase (*atpA*, *atpB*, *atpF*, *atpI*), NADH dehydrogenase (*ndhA*, *ndhF*, *ndhH*), cytochrome b/f complex (*petB*, *petD*), photosystem I and II (*psaA*, *psaB*, *psbA*, *psbB*, *psbC*, *psbD*), and RuBisCO large subunit (*rbcL*) proteins was undertaken using NZYSpeedy qPCR Green Master Mix (NZYTech) following the instructions provided, with a 50-fold diluted cDNA template. The reactions were performed in a 96-well plate, with two technical replicates in a CFX96 Touch System (Bio-Rad, Hercules, CA, USA). All the primers (Table S1) were designed from an *A. unedo* chloroplast (JQ067650.2) and 18S (AF206853.1) sequences, using Primer-BLAST (available at: http://www.ncbi.nlm.nih.gov/tools/primer-blast (accessed on 19 November 2023)). The gene expression was normalized for the 18S reference gene, and the relative expression was calculated according to the Pfaffl method [39].

### 5.4. Statistical Analysis

To compare the genotypes under different water conditions, the net CO_2_ assimilation rates, chlorophyll content, and relative gene expression were analyzed using Student’s *t*-test (*p* < 0.05) and using GraphPad Prism (v. 9.0.0 for Windows, San Diego, CA, USA). Values are given as means ± standard deviations of three biological replicates. Volcano plots were obtained using GraphPad Prism to identify statistically significant variation in gene expression between the control (WW) and treatment (WS) groups (0.5 < FC > 1.5, *p* < 0.05). To evaluate the interaction and significance of the evaluated parameters, a Principal Component Analysis (PCA), a correlation analysis, and a heatmap were constructed using the R software (version 4.2.2 R Foundation for Statistical Computing, Vienna, Austria) [40]. The heatmap was constructed using the Heatmap function and the package “ComplexHeatmap” [41]. The dendrogram within the heatmap was calculated with the Euclidean distance as a similarity measure. The PCA was performed using the prcomp function and the package “factoextra” [42], whereas the correlation analysis was applied using the function ggcor and the package “GGally” [43]. The similarity between the correlation matrices was tested using a Wilcoxon test and the r1 (measure of non-centrality), r2 (index of similarity), r3 (vector cosine angle similarity), and r4 (vector correlation) similarity tests using the allCorrelations function from the “MatrixCorrelation” package [44]. Finally, to putatively identify genes as plant performance predictors, linear regression models were calculated using R’s built-in function lm, to predict the net CO_2_ assimilation rates with gene relative expression. Jarque–Bera and Durbin–Watson tests were performed to test the normality and independence of the residuals, respectively.

## Data Availability

The data presented in this study are available in Supplementary Tables (Tables S2 and S3).

## Supplementary Materials

The following supporting information can be downloaded at https://www.mdpi.com/article/10.3390/plants12244133/s1, Table S1: Primer sequence from 16 *Arbutus unedo* chloroplast genes and 18S reference gene; Table S2: Expression of 16 *Arbutus unedo* chloroplast genes: fold change, log2 fold change, adjusted *p*-value, and −log 10 of the adjusted *p*-value between control and drought treatment (20 days at 18% field capacity) of two genotypes (A1 and A4); Table S3: R^2^, Jarque–Bera test, Durbin–Watson test, and regression coefficients for different predictors used in simple and multiple linear regression models. *p*-values < 0.05 combined with R^2^ > 0.8 are highlighted in bold.
